# Antimicrobial Evaluation of New Pyrazoles, Indazoles and Pyrazolines Prepared in Continuous Flow Mode

**DOI:** 10.3390/ijms24065319

**Published:** 2023-03-10

**Authors:** Adam Burke, Mara Di Filippo, Silvia Spiccio, Anna Maria Schito, Debora Caviglia, Chiara Brullo, Marcus Baumann

**Affiliations:** 1 Science Centre South, School of Chemistry, University College Dublin, Dublin 4, Ireland; 2 Department of Surgical Sciences and Integrated Diagnostics (DISC), University of Genoa, 16132 Genoa, Italy; 3 Section of Medicinal Chemistry, Department of Pharmacy (DIFAR), University of Genoa, 16132 Genoa, Italy

**Keywords:** pyrazole, indazole, pyrazoline, flow chemistry, antibacterial agents, antibiotic resistance

## Abstract

Multi-drug resistant bacterial strains (MDR) have become an increasing challenge to our health system, resulting in multiple classical antibiotics being clinically inactive today. As the de-novo development of effective antibiotics is a very costly and time-consuming process, alternative strategies such as the screening of natural and synthetic compound libraries is a simple approach towards finding new lead compounds. We thus report on the antimicrobial evaluation of a small collection of fourteen drug-like compounds featuring indazoles, pyrazoles and pyrazolines as key heterocyclic moieties whose synthesis was achieved in continuous flow mode. It was found that several compounds possessed significant antibacterial potency against clinical and MDR strains of the *Staphylococcus* and *Enterococcus* genera, with the lead compound (**9**) reaching MIC values of 4 µg/mL on those species. In addition, time killing experiments performed on compound **9** on *Staphylococcus aureus* MDR strains highlight its activity as bacteriostatic. Additional evaluations regarding the physiochemical and pharmacokinetic properties of the most active compounds are reported and showcased, promising drug-likeness, which warrants further explorations of the newly identified antimicrobial lead compound.

## 1. Introduction

Over the last twenty years, the number of multidrug-resistant bacterial strains (MDR) has grown dramatically [1]. Specifically in Europe, antibiotic resistance is responsible for approximately 33,000 deaths per year, whereas in the USA 2.8 million people are victims of a bacterial infection resistant to traditional antibiotics annually [2]. Unfortunately, MDR pathogens (i.e., bacteria resistant to at least three classes of antimicrobial drugs) are becoming very common, especially in hospitals [3], and bacteria such as *Methicillin-Resistant Staphylococcus aureus* (MRSA), *Vancomycin-Resistant Enterococci* (VRE) and *Mycobacteria* MDR are now extremely difficult to treat [4].

At the same time, the amount of antibiotics administered has significantly increased [5], and consequently a rise in the level of antimicrobial resistance among pathogens has occurred. Therefore, the identification of novel chemotherapeutic entities has become an urgent worldwide need [6]. Most of the therapeutic strategies currently used in MDR treatment are based on natural product derived molecules [7] or newly discovered small molecules that are able to target multidrug resistance bacteria.

Nitrogen-containing heterocycles are widely distributed amongst the world’s bestselling drugs because of their ability to target different biological scaffolds, which thus emerged as promising new chemotypes to treat bacterial infection [8], and pyrazoles are amongst the most promising scaffolds [9]. In fact, sets of pyrazole derivatives were recently reported as possessing antimicrobial [10,11], antifungal [12,13], anti-Leishmanial [14], antiviral [15] and antimycobacterial activities [16,17].

The reduction of the pyrazole ring leads to the pyrazoline scaffold, a five-membered heterocyclic ring system that has an endocyclic double bond with two adjacent nitrogen atoms. Pyrazolines are noted for the stability of their ring system and the reactivity of several sites that permit a series of substitution reactions to take place. Pyrazoline derivatives are electron-rich compounds displaying a wide variety of biological activities, as reported recently [18,19]. The fusion of the pyrazole ring with a benzene ring produces the indazole system, a 10 π-electron heteroaromatic system characterized by three tautomeric forms generated through prototropic annular tautomerism. This tautomerism along with the aromaticity of indazoles contributes to their diverse chemical reactivity as well as their biological properties [20].

**Figure 1:**
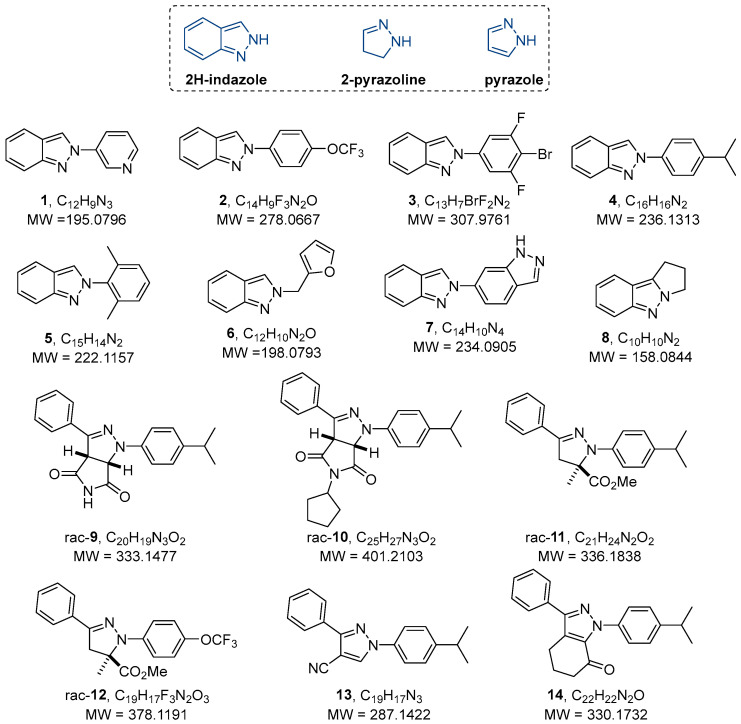
Structures of library compounds (rac = racemic; MW in g/mol).

Consequently, in addition to the well-known pyrazoles, both indazole and pyrazoline chemotypes represented interesting and privileged chemical scaffolds endowed with biological activity, including antibacterial properties [21,22]. Due to these considerations, we selected a small library of fourteen synthetic entities including indazoles, pyrazoles and pyrazolines (Figure 1) for a detailed antimicrobial evaluation. Amongst these, eight compounds were 2*H*-indazoles, including a fused tricyclic system (compound **8**), a tetrahydroindazole (compound **14**), and an unusual bis-indazole (compound **7**). The remaining structures were pyrazoline derivatives: **11** and **12** are 4,5-dihydro-1*H*-pyrazoles, whereas **9** and **10** possess a pyrazoline ring decorated with a ring-fused imide moiety.

As is visible in the above figure, all of the selected heterocyclic structures are characterized by a molecular weight below 500 g/mol, a small set of modular aryl or heteroaryl substituents, as well as a selection of further hydrogen bond donors and acceptors, which collectively adds to their drug-likeness, which is in agreement with Lipinski’s rules [23,24].

The synthesis of these pyrazole derivatives exploited continuous flow technology to streamline their accessibility. Modern drugs and their precursors are oftentimes prepared via flow-assisted routes [25,26,27,28], as this enabling technology offers several advantages over batch synthesis, including better heat and mass transfer, improved safety profiles through reactor miniaturisation, the ability to telescope reactions, and facile scalability via numbering-up or scaling-out approaches [29,30,31,32,33]. In addition, techniques that are challenging to integrate in batch mode such as photo [34,35,36,37] and electrochemistry [38,39,40] can be effectively applied in flow mode.

**Scheme 1:**
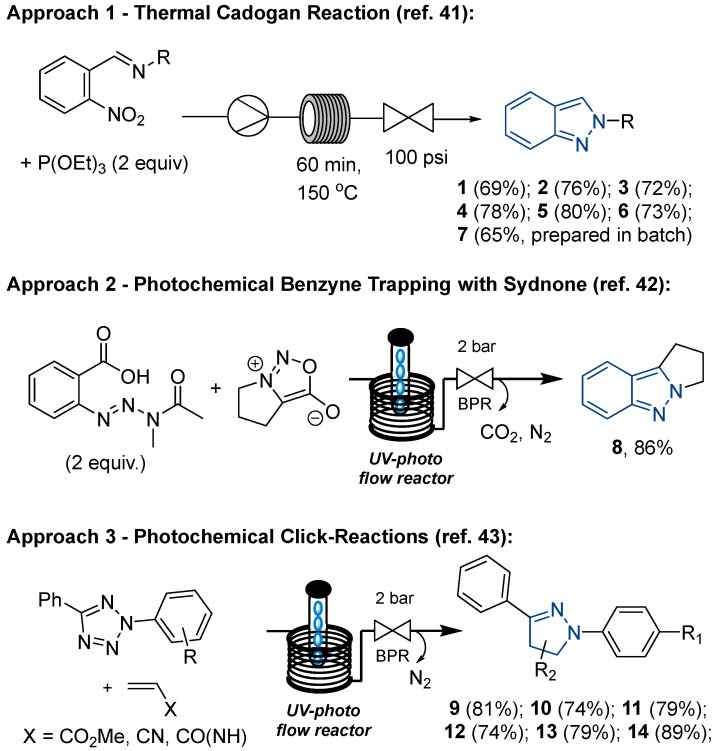
Summary of continuous flow routes towards target compounds **1**–**14** [41,42,43].

As summarised in Scheme 1, flow protocols reported previously by our group were exploited to generate the heterocyclic entities studied in this work. These included a high-temperature approach for accessing 2*H*-indazoles via a continuous Cadogan reaction [41] (compounds **1**–**7**). The tricyclic indazole scaffold **8** was accessed by a scalable photochemical flow route exploiting the in-situ formation of benzyne and its trapping with a proline derived sydnone [42]. Lastly, an interesting photochemical cycloaddition-based route converts aryl tetrazoles via nitrile imine dipoles into pyrazolines [43] (compounds **9**–**12**). The latter approach also allowed for the accessing of pyrazoles **13** and **14** that resulted from subsequent aerobic oxidation of the selected pyrazolines. All of these flow approaches have in common that the desired heterocyclic targets can be accessed in good to excellent yields in a highly reproducible manner.

## 2. Results

### 2.1. Antimicrobial Activity

The antibacterial activity of compounds **1**–**14** was tested by calculating the minimal inhibitory concentration (MIC) values on a total of eighteen clinical strains, including MDR isolates, of which twelve belonged to Gram-positive species such as *Staphylococcus aureus* (three strains), *Staphylococcus epidermidis* (three strains), *Enterococcus faecalis* (three strains) and *Enterococcus faecium* (three strains), and six Gram-negative species such as *Escherichia coli* (three strains) and *Pseudomonas aeruginosa* (three strains). Compounds were considered poorly active against a specific strain when MIC values higher than 128 µg/mL were observed. As reported in Table 1, only indazoles **2**, **3** and **5**, and pyrazoline **9** displayed interesting antibacterial activity, showing an MIC value equal to or lower than 128 µg/mL. Table 1 shows MIC values (expressed as µg/mL) obtained for the most active compounds (values reported in red) and for reference antibiotics (R.A.).

In detail, compounds **2**, **3**, **5** and **9** were found to be inactive against Gram-negative species, but active towards Gram-positive isolates.

Compound **9** showed the best antibacterial profile, displaying very low MIC values (4 μg/mL) against all of the selected isolates, as well as the MDR variants of the clinically relevant Gram-positive species of *S. aureus, S. epidermidis, E. faecalis* and *E. faecium*. It is worthy of note that the potency of compound **9** proved to be very uniform across all strains of the different species tested and irrelevant to the drug resistance patterns possessed by the various isolates.

Regarding indazole derivatives, compounds **2** and **3** showed some antibacterial activity, especially against strains of the *E. faecalis* species, while compound **5** showed a good inhibitory profile against *S. aureus* and *S. epidermidis* species, with MIC values ranging from 64 to 128 µg/mL.

Overall, compound **9**, characterized by a pyrazoline ring decorated with a ring-fused imide moiety, was identified as the most active and promising molecule, surpassing indazole derivatives **2**, **3,** and **5**.

**Figure 2:**
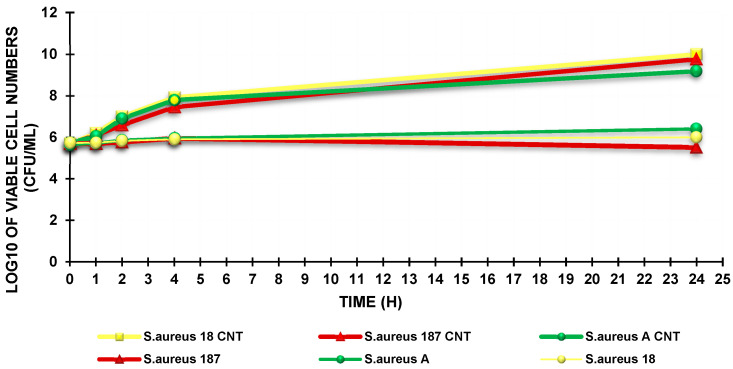
Time-killing curves performed with compound **9** at a concentration of 4× MIC on *S. aureus* 18 (MRSA) *S. aureus* 187 (MRSA), and *S. aureus* A (MRSA).

In order to investigate whether the mechanism of action of this specific compound (**9**) is based on bacteriostatic or bactericidal effects on *S. aureus*, one of the most relevant Gram-positive species in daily clinical practice, time-kill experiments were conducted on each strain of *S. aureus* considered in this study. The experiments were performed at concentrations of 4× MIC values on the 3 MRSA strains (18, 188 and A) selected for the study, and the results (Figure 2) clearly indicate that compound **9** is bacteriostatic, as it was able to maintain the concentration of the initial bacterial inoculum virtually unchanged for the 24 h of the study. Importantly, the bacteriostatic potency of **9** was uniform on all three strains tested, as seen in the very similar trends of the time-killing curves obtained.

### 2.2. Pharmacokinetic Properties and Drug-Likeness Prediction

Based on the antimicrobial activity profile, the pharmaceutical relevance of the four most active compounds (**2**, **3**, **5** and **9**), their pharmacokinetics properties, as well as their drug-likeness, were calculated by SwissADME (University of Lausanne, Switzerland) [44,45,46], and the results are reported in Table 2.

As shown above, the in silico study predicted good drug-like and pharmacokinetic properties for these compounds, particularly regarding the physicochemical properties of lipophilicity and water solubility. In detail, the fraction Csp3 is between 0 and 0.25, LogP values are between 3.79 and 4.41, the number of H bond acceptors are between 1 and 3, the number of H bond donors are between 0 and 1, and topological polar surface area (TPSA) is between 17 and 61 Å2. The TPSA of a molecule is defined as the surface sum over all polar atoms (particularly oxygen and nitrogen, including their attached hydrogen atoms) and in medicinal chemistry research is used to predict the ability of small molecules to permeate cell membranes. In general, molecules with TPSA greater than 140 Å2 are considered unable to permeate lipophilic barriers such as bacterial cell walls. The predicted TPSA values of compounds 2, 3, 5 and 9 demonstrated a good permeation capacity of these barriers. It should be noted that compound 9 is the one with the highest TPSA value (61.77 Å2), and this data could also be correlated with its greater biological activity, particularly against gram positive species with respect to the derivatives 2, 3 and 5.

Interestingly, except for compound **3,** no violations of the Lipinski rules were detected, and neither were any pan assay interference compound (PAINS) alerts found. All compounds were predicted as moderately soluble and able to penetrate the blood-brain barrier (BBB) without being substrates for Pgp. According to the calculations, the tested derivatives may inhibit some cytochrome (CYP) isoforms (1A2, 2C19, 2C9, 2D6, 3A4); in particular, compound **9** may act as an inhibitor of the CYP3A4 isoform, which warrants further study.

## 3. Discussion

**Figure 3:**
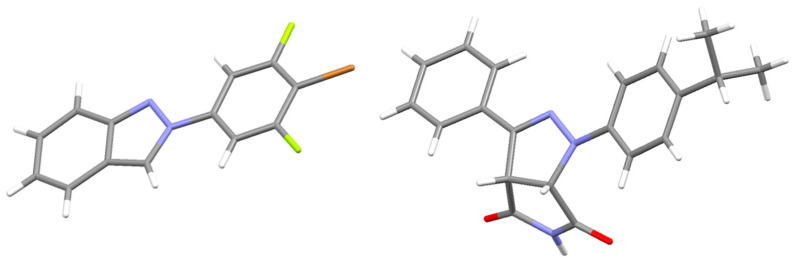
X-ray structures of indazole **3** (CCDC-2237067, **left**) and pyrazoline **9** (CCDC-2221468, **right**).

Amongst the eight 2*H*-indazoles tested in this study, three compounds (**2**, **3** and **5**) displayed weak to modest activity against different Gram-positive clinical isolates. Compounds **2** and **3** showed MIC values of around 128 μg/mL, specifically on strains of the *E. faecalis* species, while compound **5** displayed a wider spectrum of action, showing MIC values ranging from 64 to 128 μg/mL on both staphylococcal species evaluated in this study, i.e., *S. epidermidis* and *S. aureus***,** including MDR strains. On the contrary, the other 2*H*-indazoles were found to be inactive. It was found that the substitution on the *N*-aryl ring is tolerated in the ortho, meta, and para positions. As the trihalogenated compound **3** is a crystalline material, X-ray diffraction [47] was used to confirm its structure, which indicates coplanarity of the ring systems (Figure 3), which is also anticipated for compound **2**. On the other hand, compound **5** is expected to display the twisting of the ring systems due to the sterically demanding 2,6-dimethylbenzene system. The weak activity of these three species may point towards a lack of crucial binding groups including H-bond donors; however, due to the small size of these molecules, further structural editing will allow for second-generation compounds that may possess improved antimicrobial activities.

Amongst the pyrazolines, compound **9** clearly stands out for its antibacterial activity, displaying excellent potency against the most relevant clinical species of the genus *Staphylococcus*, such as *S. aureus* and *S. epidermidis*, and of the genus *Enterococcus*, such as *E. faecalis* and *E. faecium*, including several drug-resistant variants. MIC values reported against all strains of these four species were very low (4 μg/mL), and interestingly appear to be very uniform between the two different genera. Moreover, the activity of compound **9** did not seem to depend on the resistance profiles of the tested strains to current antibiotics, thus suggesting a new mechanism of action on those species. When the 24-h mechanism of action of **9** was analyzed on *S. aureus* (the clinically most relevant species of the genus *Staphylococcus*), it was found to be bacteriostatic at 4× MIC concentrations. For all three strains tested, the inhibitory action of **9** was the highest, and also prevented replication of the strains for the full duration (24 h) of this study.

Based on these results, it can be postulated that the imide moiety that allows for strong H-bonding plays a role in this activity, as the *N*-alkylated derivative **10** was found to be inactive. The kinked structure of **9** (see X-ray structure in Figure 3) may be a further advantage, as it provides for more three-dimensionality and, in turn, bioavailability. These data suggest that compound **9** (either as racemate or individual enantiomers) may serve as a new antimicrobial lead, and together with its preferrable drug-likeness parameters, encourages further developments in this direction. Such efforts to elucidate the importance of both the *N*-aryl and imide moieties as well as potential off-target activities are currently underway in our laboratories.

Crucially, the preparation of all these compounds harnessed the benefits of modern continuous flow reactor technology, thus demonstrating the rapid generation of these entities at gram-scale, which is crucial for the further synthetic manipulations and additional testing.

## 4. Materials and Methods

### 4.1. Bacterial Species Evaluated in this Study

A total of eighteen isolates belonging to a collection of Gram-positive and Gram-negative species obtained from the School of Medicine and Pharmacy of the University of Genoa (Italy) were used in this study. All were clinical strains isolated from human specimens and identified by VITEK^®^ 2 (Biomerieux, Firenze, Italy) or the matrix-assisted laser desorption/ionization time-of-flight (MALDI-TOF) mass spectrometric technique (Biomerieux, Firenze, Italy).

Of the twelve tested Gram-positive organisms, six isolates belonged to the genus *Staphylococcus,* and this includes three *Staphylococcus aureus* strains, two of which were resistant to methicillin (MRSA) and three *Staphylococcus epidermidis* isolates, two of which were resistant to methicillin (MRSE); six strains were of the *Enterococcus* genus, and included three *Enterococcus faecalis* isolates, two of which were resistant to vancomycin (VRE) isolates (one was also resistant to teicoplanin), and three *Enterococcus faecium* strains, two of which were VRE. Among the six Gram-negative strains, three were *Escherichia coli* (and included two strains resistant to carbapenems), and three were *Pseudomonas aeruginosa* (two of which were strains resistant to carbapenems and one also to colistin).

### 4.2. Determination of the Minimal Inhibitory Concentrations (MICs)

The antimicrobial activity of all fourteen compounds was assessed, and for the active compounds (**2**, **3**, **5** and **9**), their MIC values were calculated following the microdilution procedures detailed by the European Committee on Antimicrobial Susceptibility Testing EUCAST [48]. Briefly, overnight cultures of bacteria were diluted to yield a standardized inoculum of 1.5 × 10^8^ CFU/mL. Serial 2-fold dilutions of solutions of all the compounds (solubilized in DMSO), ranging from 1 to 256 µg/mL, were prepared in 96-well microplates, while DMSO not containing the tested substances was also used as a control to verify the absence of antibacterial activity of the solvent used for the experiments. Aliquots of each bacterial suspension were added to the microplates containing the above-mentioned serial two-fold dilutions of each compound to yield a final concentration of about 5 × 10^5^ cells/mL. The plates were then incubated at 37 °C. After 24 h of incubation at 37 °C, the lowest concentration of each compound that prevented a visible growth was recorded as the MIC. All MIC values were obtained in triplicate, and results were expressed reporting the modal value, which is the value that has been observed most frequently. In case of equivocal or unclear results, more than three determinations of MIC values were carried out.

### 4.3. Killing Curves

Killing curve assays for compound **9** were performed on the three isolates of *S. aureus* (two of which were MRSA) selected for the study, as previously reported [49,50].

A mid logarithmic phase bacterial culture was diluted in Mueller–Hinton (MH) broth (Merck, Darmstadt, Germany) (10 mL) containing 4× MIC of compound **9** to give a final inoculum of 3.0 × 10^5^ CFU/mL. The same inoculum was added to MH broth as a growth control. Tubes were incubated at 37 °C with constant shaking for 24 h. Samples of 0.20 mL from each tube were removed at 0, 1, 2, 4, and 24 h, diluted appropriately with a 0.9% sodium chloride solution to avoid carryover of compound **9** being tested, plated onto MH plates, and incubated for 24 h at 37 °C. Growth controls were run in parallel. The percentage of surviving bacterial cells was determined for each sampling time by comparing colony counts with those of standard dilutions of the growth control. Results have been expressed as log_10_ of viable cell numbers (CFU/mL) of surviving bacterial cells over a 24 h period. All time-kill curve experiments were performed in triplicate.

### 4.4. Preparation of Compounds Used in This Study and Synthetic Methods

The heterocyclic compounds studied in this report were prepared in accordance with recently published synthetic procedures [41,42,43]. The following is the characterisation of most active compounds:

2-(4-(Trifluoromethoxy)phenyl)-2*H*-indazole, **2**: Appearance: beige solid. Isolated yield: 76% (1.06 g, 3.8 mmol).

^1^H-NMR (400 MHz, CDCl_3_): δ/ppm 8.37 (d, *J* = 1.0 Hz, 1H), 7.96–7.90 (m, 2H), 7.76 (dd, *J* = 8.8, 1.0 Hz, 1H), 7.72–7.66 (m, 1H), 7.39–7.35 (m, 2H), 7.32 (ddd, *J* = 8.8, 6.5, 1.1 Hz, 1H), 7.15–7.08 (m, 1H). ^13^C-NMR (100 MHz, CDCl_3_): δ/ppm 150.0 (C), 148.4 (C), 139.0 (C), 127.2 (CH), 122.9 (C), 122.8 (2CH), 122.2 (2CH), 122.1 (CH), 120.4 (CH), 120.4 (CH), 120.4 (q, *J* = 254 Hz, CF_3_), 117.9 (CH). ^19^F-NMR (376 MHz, CDCl_3_): δ/ppm −58.0 (s). IR (neat) ν/cm^−1^: 3133 (w), 3063 (w), 1631 (w), 1520 (m), 1507 (m), 1383 (w), 1263 (m), 1213 (s), 1196 (s), 1155 (s), 1106 (s), 1043 (m), 951 (m), 920 (m), 845 (m), 777 (s), 753 (s), 659 (w), 533 (m), 505 (m). HRMS (Q-TOF) calculated for C_14_H_10_F_3_N_2_O 279.0740, found 279.0739 (M+H+).

2-(4-Bromo-3,5-difluorophenyl)-2*H*-indazole, **3**: Appearance: beige solid. Isolated yield: 72% (440 mg, 1.4 mmol).

^1^H-NMR (400 MHz, CDCl_3_): δ/ppm 8.37 (s, 1H), 7.74 (d, *J* = 8.8 Hz, 1H), 7.70–7.65 (m, 1H), 7.63–7.59 (m, 2H), 7.34 (dd, *J* = 8.5, 6.9 Hz, 1H), 7.13 (dd, *J* = 8.6, 6.5 Hz, 1H). ^13^C-NMR (100 MHz, CDCl_3_): δ/ppm 160.4 (2CF, dd, *J* = 249, 6 Hz), 150.2 (C), 140.8 (C), 127.9 (CH), 123.4 (CH), 123.1 (C), 120.4 (CH), 120.2 (CH), 118.0 (CH), 104.5 (2CH, dd, *J* = 28, 3 Hz), 96.8 (C, t, *J* = 25 Hz). ^19^F-NMR (376 MHz, CDCl_3_): δ/ppm −102.3 (m). IR (neat) ν/cm^−1^: 3135 (w), 3101 (w), 2921 (w) 1607 (m), 1521 (m), 1491 (s), 1446 (m), 1250 (w), 1203 (w), 1147 (w) 1024 (m), 991 (w), 861 (w), 827 (w), 751 (s). HRMS (Q-TOF) calculated for C_13_H_8_F_2_BrN_2_ 308.9833, found 308.9836 (M+H+). Crystal data (CCDC-2237067): P21/c; a 5.83880(10) b 12.60550(10) c 15.24050(10), α = 90°, β = 91.1760(10)°, γ = 90°.

2-(2,6-Dimethylphenyl)-2*H*-indazole, **5**: Appearance: yellow oil. Isolated yield: 80% (890 mg, 4.0 mmol).

^1^H-NMR (500 MHz, CDCl_3_): δ/ppm 8.00 (s, 1H), 7.84 (d, *J* = 8.7 Hz, 1H), 7.78 (d, *J* = 8.4 Hz, 1H), 7.30–7.40 (m, 2H), 7.15–7.23 (m, 3H), 2.02 (s, 6H). ^13^C-NMR (125 MHz, CDCl_3_): δ/ppm 149.2 (C), 139.8 (C), 135.6 (2C), 129.4 (CH), 128.2 (2CH), 126.2 (CH), 124.5 (CH), 122.1 (CH), 121.9 (C), 120.4 (CH), 118.1 (CH), 17.2 (2CH_3_). IR (neat) ν/cm^−1^: 3058 (m), 2922 (w), 1629 (w), 1518 (s), 1484 (m), 1388 (m), 1186 (m), 954 (m), 776 (s), 758 (s), 734 (m), 441 (m). HRMS (Q-TOF) calculated for C_15_H_15_N_2_ 223.1230, found 223.1230 (M+H+).

1-(4-Isopropylphenyl)-3-phenyl-3a,6a-dihydropyrrolo [3,4-c]pyrazole-4,6(1*H*,5*H*)-dione, **9**: Yellow solid, 81% (3.2 g, 9.6 mmol). ^1^H NMR (CDCl_3_, 400 MHz): δ/ppm 8.43 (s, 1H), 7.99 (d, *J* = 7.1 Hz, 2H), 7.46 (d, *J* = 8.7 Hz, 1H), 7.44–7.36 (m, 3H), 7.20 (d, *J* = 8.6 Hz, 2H), 5.11 (d, *J* = 10.9 Hz, 1H), 4.85 (d, *J* = 10.9 Hz, 1H), 2.87 (hept, *J* = 6.9 Hz, 1H), 1.23 (d, *J* = 7.0 Hz, 6H).

^13^C NMR (CDCl_3_, 100 MHz): δ/ppm = 172.6 (C), 171.5 (C), 142.5 (C), 142.3 (C), 142.1 (C), 130.3 (C), 129.4 (CH), 128.6 (2CH), 127.1 (2CH), 127.0 (2CH), 114.4 (2CH), 66.9 (CH), 54.6 (CH), 33.4 (CH), 24.1 (CH_3_), 24.1 (CH_3_). IR (neat): 3255 (broad), 2959 (m), 2869 (w), 1784 (s), 1610 (w), 1514 (s), 1381 (m), 1342 (m), 1207 (m), 1192 (m), 827 (m), 736 (m) cm^–1^. HRMS (ESI+): m/z [M+H]+ calcd for C_20_H_20_N_3_O_2_: 334.1550; found: 334.1551. Crystal data (CCDC-2221468): P21/c; a 17.3845(5) b 6.2983(2) c 15.8490(3), α = 90°, β = 100.537(2)°, γ = 90°.

Solvents were purchased from Sigma–Aldrich and Fisher Scientific, and used without further purification. Substrates and reagents were purchased from Alfa Aesar, Fisher Scientific, Fluorochem, or Sigma–Aldrich, and used as received. ^1^H NMR spectra were recorded with 400 and 500 MHz instruments and are reported relative to residual solvent: CHCl3 (δ = 7.26 ppm). ^13^C NMR spectra were recorded with the same instruments (100 and 125 MHz) and again are reported relative to CHCl_3_ (δ = 77.16 ppm). Data reported for 1H NMR are as follows: chemical shift (δ/ppm) (multiplicity, coupling constant (Hz), integration). Multiplicities are reported as follows: s = singlet, d = doublet, t = triplet, q = quartet, p = pentet, h = heptet, m = multiplet. Data for ^13^C{1H} NMR are reported in terms of chemical shift (δ/ppm) and multiplicity (C, CH, CH_2_, or CH_3_). COSY, HSQC and HMBC, experiments were used in the structural assignment. IR spectra were recorded with a Bruker Platinum spectrophotometer (neat, ATR sampling) with the intensities of the characteristic signals being reported as weak (w, <20% of the tallest signal), medium (m, 21−70% of the tallest signal), or strong (s, >71% of the tallest signal). High-resolution mass spectrometry (HRMS) was performed using the indicated techniques with a micromass LCT orthogonal time-of-flight mass spectrometer with leucine-enkephalin (Tyr-Gly-Phe-Leu) as an internal lock mass. For UV/Vis measurements, a Shimadzu UV-1800 UV spectrophotometer was used. Continuous-flow experiments were performed with a Vapourtec E-Series system equipped with a UV150 photoreactor in combination with a high-power LED emitting light at 365 nm wavelength and a medium-pressure Hg-lamp (combined with a low-pass filter).

## 5. Conclusions

In summary, we report on the identification of new indazole and pyrazoline derivatives that possess promising antimicrobial activities, particularly against gram positive bacteria. The most promising lead compound **9** is characterized by a bicyclic pyrazoline scaffold featuring an imide moiety. Significant activity against several drug-resistant staphylococcus and enterococcus strains was found for this species, reaching MIC values of 4 mg/mL. Physicochemical and pharmacokinetic data was collected using Swiss ADME as a tool indicating that the four most active structures (**2**, **3**, **5** and **9**) display good drug-likeness properties. The synthesis of all compounds was facilitated by continuous flow approaches to streamline and expedite the generation of these azacyclic structures. Our results showcase the value of screening synthetic small molecules to identify new antibiotic lead compounds whose further studies are currently underway in our laboratories. Additional studies are necessary to identify the exact mechanism of action of this class of molecules.

## Figures and Tables

**Table 1:** MIC values of active compounds against clinically relevant isolates of Gram-positive and Gram-negative species and those of reference antibiotics expressed in µg/mL.

Strains	*MIC* (μg/mL)				
	2	3	5	9	R.A.
S. aureus 18 *	>128	>128	128	4	256 (O)
***S. aureus 187***	>128	>128	128	4	0.5 (O)
S. aureus A *	>128	>128	*64*	*4*	256 (O)
S. epidermidis 22 *	>128	>128	*128*	*4*	16 (O)
***S. epidermidis 180***	>128	>128	*128*	*4*	0.25 (O)
S. epidermidis 2R *	>128	>128	*64*	*4*	256 (O)
***E. faecalis 1 °^,#^***	*128*	*128*	>128	*4*	64 (V); 512 (T)
***E. faecalis 50 °***	>128	128	>128	*4*	32 (V)
***E. faecalis 365***	*128*	*128*	>128	*4*	1 (V)
***E. faecium 300 °***	>128	>128	>128	*4*	256 (V)
***E. faecium 364 °***	>128	>128	>128	*4*	256 (V)
***E. faecium 503***	>128	>128	>128	*4*	1 (V)
***E. coli 224***	>128	>128	>128	>128	0.125 (I)
E. coli 462 **	>128	>128	>128	>128	16 (I)
E. coli 477 **	>128	>128	>128	>128	16 (I)
P. aeruginosa 1V **	>128	>128	>128	>128	32 (I)
P. aeruginosa 265 **^,^°°	>128	>128	>128	>128	16 (I): 16 (C)
***P. aeruginosa 403***	>128	>128	>128	>128	1 (I)

Experiments were carried out at least in triplicate; * indicates resistance towards methicillin; ° indicates resistance to vancomycin; # indicates resistance to teicoplanin; ** indicates resistance to imipenem; °° indicates resistance to colistin. R.A. (reference antibiotics) were: oxacillin (O), vancomycin (V), teicoplanin (T), imipenem (I) and colistin (C). Only compounds with MIC values equal or lower than 128 µg/mL are reported (data in red).

**Table 2:** Predicted pharmacokinetic and drug-like properties of selected compounds.

	2	3	5	9
**Physicochemical** **Property**				
MW (g/mol)	278.23	309.11	222.29	333.38
Fraction Csp^3^	0.07	0.00	0.13	0.25
Rotatable bonds	3	1	1	3
H-bond acceptors	5	3	1	3
H-bond donors	0	0	0	1
TPSA ^a^ (Å^2^)	27.05 Å^2^	17.82 Å^2^	17.82 Å^2^	61.77 Å^2^
**Lipophilicity**				
LogP ^b^	4.41	4.12	3.96	3.79
**Water solubility**				
Solubility (mg/mL) ^c^	0.00555 mg/mL	0.00387 mg/mL	0.0111 mg/mL	0.0118 mg/mL
Solubility	moderately	moderately	moderately	moderately
**Pharmacokinetics**				
GI absorption	high	high	high	high
BBB permeant	yes	yes	yes	yes
Pgp substrate	no	no	no	no
CYP1A2 inhibitor	yes	yes	yes	no
CYP2C19 inhibitor	yes	yes	yes	yes
CYP2C9 inhibitor	no	no	no	yes
CYP2D6 inhibitor	yes	no	yes	no
CYP3A4 inhibitor	no	no	no	yes
**Druglikeness**				
Lipinski violations	0	1 violation: MLogP > 4.15	0	0
**Medicinal chemistry**				
PAINS alerts	0	0	0	0
Brenk alerts [46]	0	1 (halogenated ring)	0	1 (phthalimide)

^a^ Topological Polar Surface Area. ^b^ Predicted according to XLOGP3 program. ^c^ Values predicted by ESOL method [45].

## Data Availability

All data supporting the reported results are included in the present manuscript.
